# Intraoperative hypotension associated with postoperative acute kidney injury in hypertension patients undergoing non-cardiac surgery: a retrospective cohort study

**DOI:** 10.1093/burnst/tkae029

**Published:** 2024-07-24

**Authors:** Jin Li, Yeshuo Ma, Yang Li, Wen Ouyang, Zongdao Liu, Xing Liu, Bo Li, Jie Xiao, Daqing Ma, Yongzhong Tang

**Affiliations:** Department of Anesthesiology, Third Xiangya Hospital, Central South University, 138 Tongzipo Road, Changsha, 410013, China; Department of Geriatrics, Third Xiangya Hospital, Central South University, 138 Tongzipo Road, Changsha, 410013, China; Department of Anesthesiology, Third Xiangya Hospital, Central South University, 138 Tongzipo Road, Changsha, 410013, China; Department of Anesthesiology, Third Xiangya Hospital, Central South University, 138 Tongzipo Road, Changsha, 410013, China; Department of Geriatrics, Third Xiangya Hospital, Central South University, 138 Tongzipo Road, Changsha, 410013, China; Department of Anesthesiology, Third Xiangya Hospital, Central South University, 138 Tongzipo Road, Changsha, 410013, China; Operation Center, Third Xiangya Hospital, Central South University, 138 Tongzipo Road, Changsha, 410013, China; Department of Emergency, Third Xiangya Hospital, Central South University, 138 Tongzipo Road, Changsha, 410013, China; Division of Anesthetics, Pain Medicine & Intensive Care, Department of Surgery and Cancer, Chelsea and Westminster Hospital, Imperial College London, South Kensington Campus, London SW7 2AZ, UK; Department of Anesthesiology, Third Xiangya Hospital, Central South University, 138 Tongzipo Road, Changsha, 410013, China; Clinical Research Center, Third Xiangya Hospital, Central South University, 138 Tongzipo Road, Changsha, 410013, China

**Keywords:** Acute kidney injury, Intraoperative hypotension, Non-cardiac surgery, Chronic hypertension

## Abstract

**Background:**

Acute kidney injury (AKI) is a common surgical complication and is associated with intraoperative hypotension. However, the total duration and magnitude of intraoperative hypotension associated with AKI remains unknown. In this study, the causal relationship between the intraoperative arterial pressure and postoperative AKI was investigated among chronic hypertension patients undergoing non-cardiac surgery.

**Methods:**

A retrospective cohort study of 6552 hypertension patients undergoing non-cardiac surgery (2011 to 2019) was conducted. The primary outcome was AKI as diagnosed with the Kidney Disease-Improving Global Outcomes criteria and the primary exposure was intraoperative hypotension. Patients’ baseline demographics, pre- and post-operative data were harvested and then analyzed with multivariable logistic regression to assess the exposure–outcome relationship.

**Results:**

Among 6552 hypertension patients, 579 (8.84%) had postoperative AKI after non-cardiac surgery. The proportions of patients admitted to ICU (3.97 vs. 1.24%, *p* < 0.001) and experiencing all-cause death (2.76 vs. 0.80%, *p* < 0.001) were higher in the patients with postoperative AKI. Moreover, the patients with postoperative AKI had longer hospital stays (13.50 vs. 12.00 days, *p* < 0.001). Intraoperative mean arterial pressure (MAP) < 60 mmHg for >20 min was an independent risk factor of postoperative AKI. Furthermore, MAP <60 mmHg for >10 min was also an independent risk factor of postoperative AKI in patients whose MAP was measured invasively in the subgroup analysis.

**Conclusions:**

Our work suggested that MAP < 60 mmHg for >10 min measured invasively or 20 min measured non-invasively during non-cardiac surgery may be the threshold of postoperative AKI development in hypertension patients. This work may serve as a perioperative management guide for chronic hypertension patients.

**Trial registration:**

clinical trial number: ChiCTR2100050209 (8/22/2021).

http://www.chictr.org.cn/showproj.aspx?proj=132277.

Highlights

- We explored the appropriately perioperative management of intraoperative blood pressure to prevent against postoperative acute kidney injury (AKI) in the hypertensive patients undergoing a non-cardiac surgery.
- MAP less than 60 mmHg for more than 20 min was an independent risk factor of postoperative AKI, and for more than 10 min in the subgroup of invasive MAP.
- Our results provided the important evidences for the rational management of intraoperative MAP in the hypertensive patients. It also suggested that invasive measurement could reflect the true values of blood pressure more exactly.

## Background

Acute kidney injury (AKI) is a common surgical complication. Previous studies have shown that surgery-related AKI occurs in ~18.3% of hospitalized patients [1], up to 74% of critically ill patients [2,3], 8.2–15.1% of patients undergoing noncardiac surgery [4,5] and 36–45% of patients undergoing cardiac surgery [6,7]. Importantly, postoperative AKI not only prolongs length of hospital stay but also leads to chronic kidney disease in some patients, which requires long-term renal replacement therapy and causes a heavy economic burden [8,9]. Perioperative hypotension was reported to be most prevalent during the intraoperative period. Patients who had hypertension or dyslipidemia were more likely to experience hypotension intraoperatively [10], and advanced age, high American Society of Anesthesiologists (ASA) score, anesthesia method and surgical position were also risk factors for intraoperative hypotension [11,12]. Thus, intraoperative hypotension may be an important cause of postoperative AKI [13]. The morbidity of postoperative AKI decreases with active management of intraoperative blood pressure [14,15]. Therefore, it is urgent to explore the appropriate perioperative management of intraoperative blood pressure to prevent postoperative AKI in patients undergoing noncardiac surgery.

Previous studies demonstrated that intraoperative hypotension was associated with postoperative AKI [14,16,17]. Indeed, an intraoperative mean arterial pressure (MAP) < 60 mmHg for 11–20 min and <55 mmHg for >10 min was associated with postoperative AKI in patients who underwent noncardiac surgery [14]. Our previous report also suggested that the risk of postoperative AKI increased significantly in patients who underwent noncardiac surgery when their intraoperative MAP was <55 mmHg for >10 min [15]. However, the threshold of intraoperative hypotension may not be useful for hypertensive patients whose autonomic regulation function curve of renal blood flow often shifts to the right [18–20]. Chronic hypertension is one of the most common chronic diseases, affecting 1.4 billion patients worldwide, and a management guide for intraoperative MAP in these patients is not yet available [20,21]. Therefore, we conducted a retrospective cohort study to investigate the relationships between various levels of intraoperative MAP and total duration and postoperative AKI in hypertensive patients who underwent noncardiac surgery.

## Methods

### Study design and cohort selection

**Figure 1:**
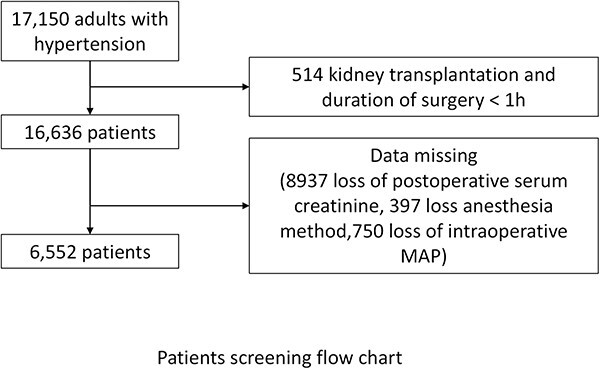
Flowchart of patients selection

This retrospective cohort study of hypertensive patients who underwent noncardiac surgery from December 2011 to December 2019 was approved by the Ethics Committee (R21044) of Third Xiangya Hospital, Central South University, Hunan, China. The requirement for written informed consent was waived. The enrollment criteria included hypertensive patients who underwent noncardiac surgery at first admission and were aged ≥18 years. Patients with the following conditions were excluded: kidney transplantation, duration of surgery <60 min, loss of postoperative serum creatinine and loss of 30% of perioperative variables in a single patient (including loss of anesthesia and loss of intraoperative MAP). Ultimately, 6552 hypertensive patients were enrolled in the study, see Figure 1. Clinical trial number: ChiCTR2100050209 (8/22/2021). This manuscript adheres to the applicable STROBE guidelines.

### Primary and secondary outcomes

The primary outcome was AKI diagnosed with the Kidney Disease-Improving Global Outcomes criteria [22], namely, a 0.3 mg/dl increase in creatinine over the preoperative value during the first two postoperative days or >1.5-fold increase during the first seven postoperative days. The secondary outcomes included intensive care unit (ICU) admission, length of hospital stay and all-cause death.

### Intraoperative blood pressure

The intraoperative MAP was extracted from the electronic medical records. When an arterial catheter was used, MAP was recorded at 30 s intervals. Without the arterial catheter line, noninvasive blood pressure was measured at 5 min intervals. In our study, blood pressure measured with an arterial catheter was recorded in 2787 patients (42.54%). Blood pressure values between measurements were linearly interpolated. According to our previous study [15], MAP ≤60 mmHg was defined as hypotension, and the total length of intraoperative hypotension was calculated.

### Data sources

A formal sample size calculation was not performed because the effect sizes were unknown during the time of study design. Demographics and perioperative data, including diagnosis, blood pressure, complications, laboratory examinations, preoperative medicines, types of surgery, intraoperative conditions and postoperative outcomes, were extracted from the electronic medical records with Lex Clinical Research Data Warehouse Software (Le9 Healthcare Technology, Shanghai, China). All the data were also checked manually to ensure completeness and accuracy.

### Statistical analysis

We performed multiple imputation to recover missing data (Table S1, see online supplementary material). Continuous variables are expressed as the mean ± SD if normally distributed; otherwise, non-normally distributed data are expressed as the median (interquartile range). The data was analyzed by Student’s t test or Kruskal–Wallis rank-sum test followed by the least significant difference *post hoc* test wherever appropriate. Categorical variables are presented as numbers with proportions and were analyzed by the chi-square test. Bivariate analyses were used to compare the characteristics of the cohort with and without intraoperative hypotension for different MAP thresholds. Using line and curve fitting (restricted cubic spline), a preliminary analysis of the relationship between MAP exposure level and cumulative time during surgery and postoperative AKI was performed. Univariable logistic regression and collinearity analysis were conducted to select the variables that were significant at *p* < 0.05 and had a variance expansion factor < 4. We then performed multivariate logistic regression analysis to calculate the odds ratio of postoperative AKI for different durations of operation (0, 1–5, 6–10, 11–20, >20 min) with MAP ≤60 mmHg. To assess the robustness of our findings, a sensitivity verification was performed in five subgroups (general anesthesia, invasively measured MAP, without diabetes, estimated glomerular filtration rate (eGFR) > 60 ml/min/1.73 m^2^, primary hypertension) and reported as adjusted odds ratios and associated 95% confidence intervals and *p* values. The data were analyzed by using SAS version 9.4 software (SAS Institute, Inc., Cary, NC, USA) and CRAN R (v3.4.1). A two-tailed *p* value < 0.05 was considered to indicate statistical significance.

## Results

### Primary and secondary outcomes

A total of 6552 surgeries were ultimately included in this study (Figure 1), and 579 (8.84%) patients developed postoperative AKI (Table 1). For the secondary outcome, the proportions of patients admitted to the ICU (3.97 *vs* 1.24%, *p* < 0.001) and experiencing all-cause death (2.76 *vs* 0.80%, *p* < 0.001) were greater among the patients with postoperative AKI (Table 2). Moreover, the patients with postoperative AKI had longer hospital stays (13.50 *vs* 12.00 days, *p* < 0.001).

**Table 1:** Baseline characteristics of patients with and without postoperative AKI

**Primary outcome**	**No AKI (n = 5973)**	**AKI (n = 579)**	***P*** ** value**
**Baseline**			
Age (years)	61.95 ± 11.74	61.90 ± 13.26	0.654
Male, n (%)	2833 (47.43%)	326 (56.30%)	<0.001
Weight (kg)	63.90 ± 13.97	62.88 ± 12.54	0.294
Smoke	494 (8.28%)	54 (9.34%)	0.380
Alcohol	332 (5.57%)	34 (5.88%)	0.753
MAP (mmHg)	100.49 ± 9.58	101.20 ± 10.38	0.165
Hemoglobin (g/l)	123.61 ± 21.44	110.67 ± 26.38	<0.001
eGFR (ml/min/1.73 m^2^)	79.78 ± 26.31	55.19 ± 35.69	<0.001
Albumin (g/l)	39.64 ± 5.09	36.52 ± 6.80	<0.001
**Preoperative medications**			
RASI	206 (3.45%)	19 (3.28%)	0.833
Calcium antagonists	27 (0.45%)	8 (1.38%)	0.003
Diuretics	174 (2.91%)	53 (9.15%)	<0.001
β-Blockers	66 (1.10%)	18 (3.11%)	<0.001
Lipid regulating drugs	23 (0.39%)	6 (1.04%)	0.331
Anticoagulant drugs	373 (6.24%)	77 (13.30%)	<0.001
**Comorbidities**			
Diabetes	1317 (22.05%)	165 (28.50%)	<0.001
Coronary heart disease	397 (11.72%)	47 (13.35%)	0.368
Respiratory diseases	565 (9.46%)	96 (16.58%)	<0.001
Emergency	675 (11.30%)	155 (26.77%)	<0.001
Cerebrovascular diseases	171 (5.05%)	26 (7.39%)	0.062
Primary hypertension	5540 (92.75%)	474 (81.87%)	<0.001
Tumor	1767 (29.58%)	186 (32.12%)	0.202

*AKI* acute kidney injury, *MAP* mean arterial pressure, *RASI* renin–angiotensin system inhibitors, *eGFR* estimated glomerular filtration rate

**Table 2:** Intraoperative characteristics of patients with and without postoperative AKI

**Primary outcome**	**No AKI (n = 5973)**	**AKI (n = 579)**	***P*** ** value**
**ASA grade, n (%)**			<0.001
I	74 (1.24%)	1 (0.17%)	
II	2455 (41.10%)	135 (23.32%)	
III	2945 (49.31%)	316 (54.58%)	
IV	357 (5.98%)	107 (18.48%)	
V	20 (0.33%)	7 (1.21%)	
**Intraoperative**			
Duration of surgery (min)	168.43 ± 91.75	170.78 ± 99.93	0.839
In fluids (ml)	2000 (1250–3000)	1740 (1000–2800)	<0.001
Out fluids (ml)	700 (400–1000)	600 (300–900)	<0.001
Hemorrhage (ml)	100 (50–300)	200 (50–500)	<0.001
Erythrocyte transfusions (ml)	0 (0–0)	0 (0–400)	<0.001
Vasoactive drugs	1143 (19.14%)	173 (29.88%)	<0.001
General anesthesia	4222 (70.68%)	433 (74.78%)	0.038
MAP ≤ 60, n (%)			<0.001
0 min	3832 (64.2%)	329 (56.8%)	
0–5 min	1521 (25.5%)	153 (26.4%)	
5–10 min	329 (5.5%)	39 (6.7%)	
10–20 min	186 (3.1%)	32 (5.5%)	
>20 min	103 (1.7%)	26 (4.5%)	
MAP ≤ 70, n (%)			0.03
0 min	2245 (37.6%)	211 (36.4%)	
0–5 min	1453 (24.3%)	125 (21.6%)	
5–10 min	630 (10.5%)	65 (11.2%)	
10–20 min	689 (11.5%)	58 (10%)	
>20 min	934 (15.6%)	118 (20.4%)	
**Type of surgery**			<0.001
General surgery	1764 (29.53%)	139 (24.01%)	
Urological	1047 (17.95%)	143 (24.70%)	
Gynecological	652 (11.37%)	29 (5.01%)	
Orthopedic	1100 (18.58%)	79 (13.65%)	
Neurosurgical	487 (8.16%)	35 (6.05%)	
Thoracic	379 (6.35%)	29 (5.01%)	
Others	701 (11.74%)	144 (24.87%)	
**Secondary outcome**			
Transferred to ICU (%)	74 (1.24%)	23 (3.97%)	<0.001
Hospital days (days)	12.00 (8.00–17.00)	13.50 (9.00–20.50)	<0.001
All-cause death (%)	48 (0.80%)	16 (2.76%)	<0.001

*ASA* American Society of Anesthesiologists, *ICU* intensive care unit, *MAP* mean arterial pressure, *AKI* acute kidney injury

The proportions of patients who experienced postoperative AKI increased when MAP was <60 mmHg. As MAP decreased and the total duration increased, the mortality of postoperative AKI increased (Table S2, see online supplementary material).

**Figure 2:**
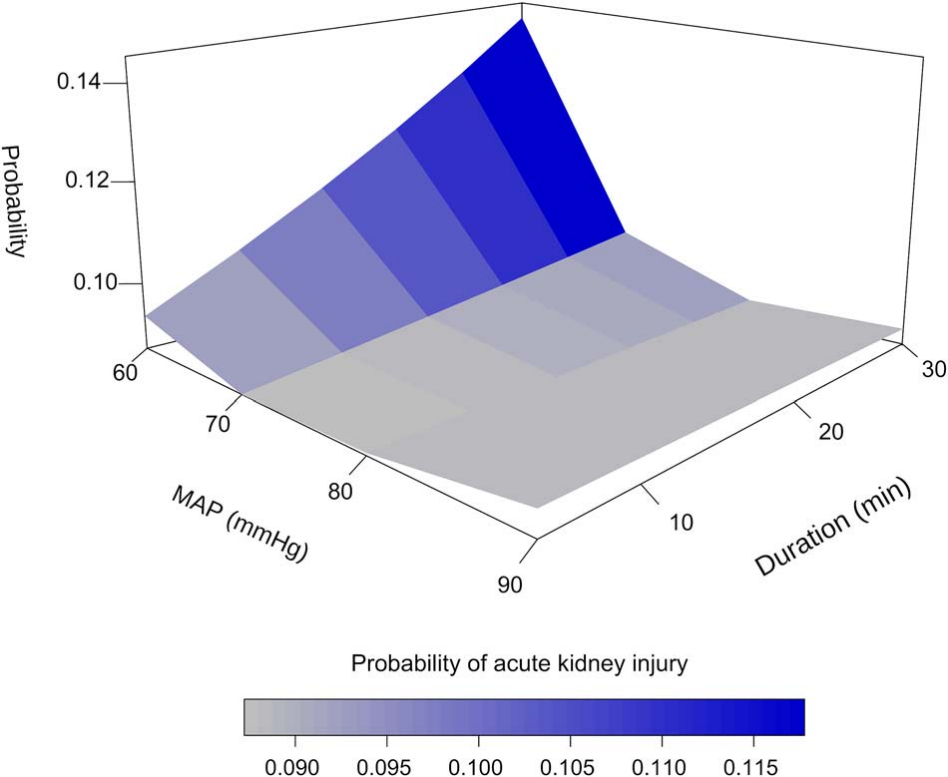
Correlation between the risk of postoperative AKI and the lower and long total duration of MAP. *AKI* acute kidney injury, *MAP* mean arterial pressure

There was a positive correlation between the risk of postoperative AKI and a shorter and longer total duration of MAP. The peak mortality of postoperative AKI was >14% when MAP was <60 mmHg for >20 min (Figure 2).

### Logistic analysis

Single-variable logistic regression analysis revealed that MAP < 60 mmHg was associated with postoperative AKI (see supplementary Table S3 in the online supplementary material). In addition, many factors, such as age, sex, basic MAP, diabetes and secondary hypertension, were associated with postoperative AKI in patients who experienced MAP < 60 mmHg (Table S3, see online supplementary material).

After adjustment for age, sex, basic MAP, smoking status, alcohol consumption, emergency surgery, American Society of Anesthesiologists (ASA) grade, preoperative medication (diuretics, renin–angiotensin system inhibitors, calcium antagonists, β-blockers), hemoglobin, albumin, alanine aminotransferase, magnitude of surgery, type of anesthesia, total duration of surgery, in/out fluids, hemorrhage and vasoactive drugs, multivariate logistic regression analysis revealed that intraoperative hypotension with an intraoperative MAP < 60 mmHg for >20 min was a risk factor for postoperative AKI (Table 3). Furthermore, before the regression analysis, collinear analysis showed that there was no multicollinearity among these adjusted factors (kappa coefficient = 15.16).

**Table 3:** Comparison of odds ratios of postoperative acute kidney injury in patients experiencing different total duration of intraoperative mean arterial pressure (MAP)

**MAP, mmHg**	**Total duration of intraoperative hypotension (min)**				
	**0**	**0–5**	**5–10**	**10–20**	**>20**
≤60	Reference	1.06 (0.85–1.32)	1.19 (0.80–1.71)	1.39 (0.89–2.12)	1.91 (1.13–3.15)^*^
≤70	Reference	0.81 (0.63–1.04)	1.05 (0.76–1.44)	0.79 (0.56–1.09)	1.03 (0.77–1.37)

Adjusted for age, sex, basic MAP, smoke, alcohol, emergency, ASA grade, preoperative medication (diuretics, renin–angiotensin system inhibitors, calcium antagonists, β-blockers), hemoglobin, albumin, alanine aminotransferase, magnitude of surgery, types of anesthesia, duration of surgery, in/out fluids amount, hemorrhage and vasoactive drugs

^*^ *p* < 0.05

Then, stepwise logistic regression was performed to further assess the data (Table 4). The logistic regression was stable, and there was a strong association between intraoperative hypotension (MAP < 60 mmHg for > 20 min) and postoperative AKI.

**Table 4:** Risk of postoperative AKI in patients experiencing different total duration of intraoperative hypotension

**Total duration of intraoperative hypotension (min)**	**AKI OR (95% CI)**		
	**Model 1**	**Model 2**	**Model 3**
0	Reference	Reference	Reference
0–5	1.17 (0.96–1.43)	1.27 (0.99–1.49)	1.06 (0.85–1.32)
5–10	1.38 (0.96–1.94)	1.44 (1.00–2.02)^*^	1.19 (0.80–1.71)
10–20	2.00 (1.33–2.92)^***^	2.10 (1.39–3.07)^***^	1.39 (0.89–2.12)
>20	2.94 (1.85–4.52)^***^	3.17 (2.00–4.90)^***^	1.91 (1.13–3.15)^*^

*AKI* acute kidney injury, *MAP* mean arterial pressure, *OR* odds ratio, *CI* confidence interval

Model 1: no adjustment. Model 2: adjusted for age, sex, basic MAP, smoke and alcohol. Model 3: adjusted for Model 2 plus diabetes, emergency, American Society of Anesthesiologists grade, preoperative medications (diuretics, renin–angiotensin system inhibitor, calcium antagonists, β-blockers), hemoglobin, albumin, alanine aminotransferase, magnitude of surgery, type of anaesthesia, duration of surgery, in/out fluids amount, erythrocyte transfusions and vasoactive drugs

^*^ *p* < 0.05,

^***^ *p*< 0.001

### Sensitivity analysis

**Figure 3:**
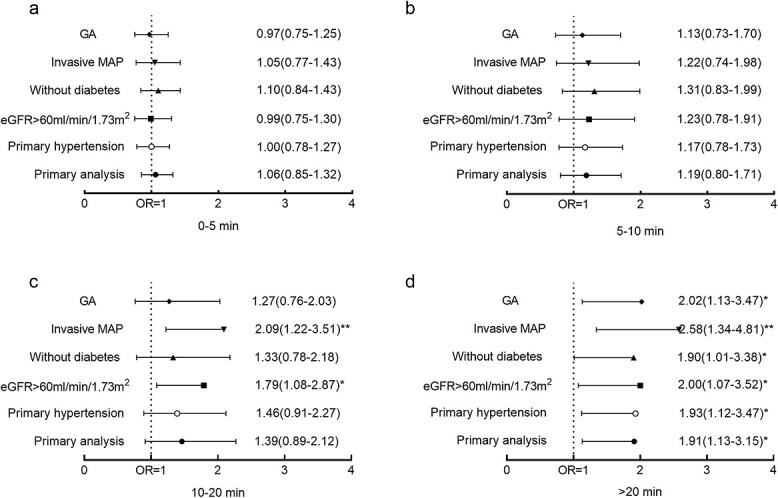
Sensitivity analyses between postoperative AKI and hypertensive patients with MAP ≤ 60 mmHg across different subgroups. (**a**) Total hypotension duration of 0-5 min. (**b**) Total hypotension duration of 5-10 min. (**c**) Total hypotension duration of 10-20 min. (**d**) Total hypotension duration of more than 20 min. *AKI* acute kidney injury, *MAP* mean arterial pressure, *GA* general anesthesia group

To further confirm the relationship between intraoperative hypotension and postoperative AKI obtained from the above analysis, sensitivity analyses were performed in different subgroups. To eliminate the risks of comorbidities, type of anesthesia and the source of record, the subgroups were set up as primary hypertension, without diabetes, eGFR > 60 ml/min/1.73 m^2^, general anesthesia and invasive MAP (Figure 3). Sensitivity analyses revealed that intraoperative hypotension (MAP < 60 mmHg for >20 min) was an independent risk factor for postoperative AKI in the subgroups of patients with primary hypertension, without diabetes and who were receiving general anesthesia. Interestingly, MAP < 60 mmHg for >10 min was an independent risk factor in the subgroups when the preoperative eGFR was >60 ml/min/1.73 m^2^ and the MAP was invasively measured; this suggested that there was a strong and stable relationship between intraoperative hypotension and postoperative AKI in hypertension patients among the different subgroups.

Finally, variance inflation factor analysis was performed (Table S4, see online supplementary material), which further suggested nonmulticollinearity among the adjusted factors in the multivariate logistic regression.

## Discussion

The results obtained in our study suggested that intraoperative hypotension with MAP < 60 mmHg for > 10 min when MAP was measured invasively and > 20 min when MAP was measured noninvasively was a significant independent risk factor for postoperative AKI in hypertensive patients who underwent noncardiac surgery. Patients who experienced postoperative AKI had an increased proportion of all-cause deaths with longer hospital stays.

Postoperative AKI is associated not only with an increase in short-term mortality [23,24] but also with a high risk of long-term complications [25]. Patients who experienced postoperative AKI were reported to be eight times more likely to die within 30 days of surgery [26]. Postoperative AKI is associated with intraoperative hypotension, which can be ameliorated with rational management of intraoperative blood pressure [14,15]. However, there is still no clear definition of the duration and severity of intraoperative hypotension that offers a rational management guide for intraoperative MAP to prevent postoperative AKI development in hypertensive patients [20], although a previous report showed that an intraoperative MAP < 60 mmHg for 11–20 min and <55 mmHg for >10 min was associated with postoperative AKI in patients who underwent noncardiac surgery [14]. Moreover, among patients who underwent noncardiac surgery and were <60 years old, our previous data suggested that the risk of postoperative AKI increased significantly when the intraoperative MAP was <55 mmHg for >10 min [15]. Furthermore, our current work suggested that an intraoperative MAP < 60 mmHg for >20 min was a significant independent risk factor for postoperative AKI in hypertensive patients who underwent noncardiac surgery, which was consistent with the fact that the autonomic regulation function curve of renal blood flow shifted to the right in chronic hypertension patients [18,19]; this requires high perfusion pressure in hypertensive patients to effectively meet the high blood-flow requirements of the kidney cortex, while low blood pressure during surgery clearly compromises the blood supply [20]. Consequently, sublethal ischemia causes tubule cell injury and subsequently triggers an inflammatory response to promote postoperative AKI development [27]. Previous studies by our team and others revealed that toll-like receptors-4 (TLR4) was increased in renal tissue after ischemia–reperfusion injury, especially in renal tubular epithelial cells and vascular endothelial cells [28,29]. TLR4 activation not only promoted the release of proinflammatory mediators (including interleukin-6, interleukin-1, and tumor necrosis factor-α) and facilitated leukocyte migration and infiltration into the renal interstitium [30] but also sustained tubular necrosis and ultimately potentiated renal fibrosis [28]. More importantly, AKI, in turn, promoted significant systemic inflammatory responses in distant organs, including the lungs, heart, liver and brain, and resulted in multiorgan failure and death [31–33].

Previous studies have suggested that continuous blood pressure monitoring reflects real-time blood pressure stability better than noninvasive blood pressure monitoring during general anesthesia during orthopedic surgery [34]. Furthermore, invasive blood pressure measurements can detect hemodynamic perturbations more effectively and in a timely manner to facilitate treatment after surgery [35]. In addition, invasive blood pressure measurements and related management are related to lower mortality in critically ill patients with chronic hypertension [36,37]. Our data and those described above suggest that hypertensive patients may benefit more from invasive measurements of MAP than noninvasive measurements in terms of postoperative AKI, and invasive blood pressure measurements should be implemented in daily clinical practice for elderly hypertensive patients.

Our study has several limitations. First, the study is a retrospective observational study; therefore, our data may not accurately reflect this group of patients. Intraoperative blood pressure modulation in patients with chronic hypertension was lower than we expected, which may be related to the large range of our blood pressure segmentation and to the mild severity of preoperative hypertension in surgical patients. Second, although a large sample of patients was collected, the present study included data from only a single center; hence, the data reported herein may not represent patient generalizability. Further multicenter data analyses are urgently needed. Third, the effects of certain high-risk factors, such as high-grade ASA and kidney dysfunction, on the relationship between intraoperative MAP and postoperative AKI were not fully elucidated, and further research is needed.

## Conclusions

In conclusion, we found that a definitive MAP of <60 mmHg lasted for >10 min when the MAP was measured invasively or >20 min when the MAP was measured noninvasively at the threshold for postoperative AKI development in hypertensive patients who underwent noncardiac surgery. Our work further suggested that invasive blood pressure monitoring should be implemented in our daily clinical practice in high-risk patients, e.g. those who are hypertensive and receive major surgery. Our work may indicate that rational intraoperative MAP management should be implemented to avoid MAPs < 60 min wherever necessary in hypertensive patients.

## Supplementary Material

Supplement_Tables_tkae029

## Data Availability

The datasets used and/or analysed during the current study are available from the corresponding author on reasonable request.

## Abbreviations

AKI: Acute kidney injury; ASA: American Society of Anesthesiologists; eGFR: Estimated glomerular filtration rate; ICU: intensive care unit; MAP: Mean arterial pressure.
