# Supplementary material for: Intraoperative hypotension associated with postoperative acute kidney injury in hypertension patients undergoing non-cardiac surgery: a retrospective cohort study
Source: Burns Trauma. 2024 Jul 24;12:tkae029. doi: 10.1093/burnst/tkae029 (PMC11267586; doi:10.1093/burnst/tkae029)
Supplement: Supplement_Tables_tkae029 [file supplement_tables_tkae029.docx]

Table S1. Interpolation of variables

| **Variable** | **Number** | **Ratio** | **Method of interpolation** |
| --- | --- | --- | --- |
| Smoke | 10 | 0.15% | Multiple imputation |
| Alcohol | 10 | 0.15% | Multiple imputation |
| Hemorrhage | 201 | 3.08% | Multiple imputation |
| In fluids amount | 1 | 0.02% | Multiple imputation |
| Preoperative alanine aminotransferase | 1143 | 17.53% | Multiple imputation |
| Preoperative albumin | 1119 | 17.16% | Multiple imputation |
| Basic MAP | 124 | 1.90% | Multiple imputation |

*MAP* mean arterial pressure

Table S2. Proportion of patients experiencing postoperative AKI, stratified by experiencing MAP ≤ 60 mmHg

| **IOH duration (min)** | **0** | **0-5** | **5-10** | **10-20** | **>20** |
| --- | --- | --- | --- | --- | --- |
| N | 4161 | 1674 | 368 | 218 | 129 |
| AKI | 7.90% | 9.10% | 10.60% | 14.70% | 20.20% |

*AKI* acute kidney injury, *IOH* intraoperative hypotension, *MAP* mean arterial pressure

Table S3. Association of perioperative characteristics with postoperative AKI in patients experiencing different intraoperative MAP

| **Variables** | **MAP ≤ 60 mmHg** | **MAP ≤ 70 mmHg** |
| --- | --- | --- |
| Age | 1.006(0.995-1.018;*P*=0.272) | 1.005(0.995-1.014;*P*=0.332) |
| Male | 1.651(1.269-2.153;*P*<0.001) | 1.855(1.493-2.309;*P*<0.001) |
| Basic MAP | 1.012(0.999-1.025;*P*=0.070) | 1.014(1.003-1.025;*P*=0.013) |
| Smoke | 1.121(0.631-1.864;*P*=0.678) | 1.271(0.856-1.830;*P*=0.214) |
| Alcohol | 1.122(0.576-1.998;*P*=0.715) | 1.321(0.823-2.026;*P*=0.223) |
| Diabetes | 1.330(0.986-1.777;*P*=0.057) | 1.479(1.163-1.869;*P*=0.001) |
| Respiratory diseases | 1.281(0.655-2.294;*P*=0.434) | 1.226(0.719-1.970;*P*=0.425) |
| Secondary hypertension | 2.823(1.883-4.145;*P*<0.001) | 2.987(2.148-4.092;*P*<0.001) |
| Emergency | 3.398(2.537-4.527;*P*<0.001) | 3.184(2.494-4.046;*P*<0.001) |
| ASA grade |  |  |
| Ⅰ&Ⅱ | Reference | Reference |
| Ⅲ | 1.822(1.299-2.598;*P*<0.001) | 1.837(1.410-2.413;*P*<0.001) |
| Ⅳ&Ⅴ | 7.094(4.770-10.649;*P*<0.001) | 6.616(4.781-9.177;*P*<0.001) |
| **Preperative** |  |  |
| Diuretics | 3.117(1.775-5.249;*P*<0.001) | 3.648(2.389-5.441;*P*<0.001) |
| RASI | 1.052(0.433-2.181;*P*=0.900) | 1.130(0.601-1.949;*P*=0.682) |
| Calcium antagonists | 1.070(0.058-5.864;*P*=0.949) | 0.843(0.046-4.297;*P*=0.870) |
| β-Blockers | 2.898(1.042-6.984;*P*=0.025) | 3.073(1.482-5.876;*P*=0.001) |
| Anticoagulant drugs | 2.300(1.493-3.450;*P*<0.001) | 2.275(1.627-3.125;*P*<0.001) |
| Hemoglobin | 1.002(1.001-1.003;*P*<0.001) | 1.002(1.001-1.002;*P*<0.001) |
| Albumin | 0.916(0.896-0.937;*P*<0.001) | 0.918(0.902-0.935;*P*<0.001) |
| Alanine aminotransferase | 1.001(1.000-1.002; *P*=0.023) | 1.001(1.000-1.002;*P*=0.006) |
| Magnitude of surgery |  |  |
| Small | Reference | Reference |
| Medium | 0.047(0.002-0.511; *P* =0.014) | 0.220(0.048-1.548; *P* =0.071) |
| Large | 0.058(0.003-0.613; *P*=0.021) | 0.301(0.069-2.058; *P* =0.142) |
| Above large | 0.063(0.003-0.667; *P*=0.025) | 0.326(0.073-2.266; *P* =0.177) |
| **Intraoperative** |  |  |
| General anesthesia | Reference | Reference |
| Spinal anesthesia | 0.979(0.618-1.490;*P*=0.923) | 0.852(0.585-1.205;*P*=0.384) |
| Compound anesthesia | 0.818(0.445-1.394;*P*=0.488) | 0.734(0.427-1.182;*P*=0.232) |
| Intravenous anesthesia | 3.414(0.487-15.947;*P*=0.144) | 1.790(0.276-6.707;*P*=0.450) |
| Nerve block | 2.133(0.483-6.782;*P*=0.243) | 1.641(0.383-4.882;*P*=0.429) |
| Duration of surgery | 1.000(0.999-1.002;*P*=0.46) | 1.001(0.999-1.002;*P*=0.335) |
| In fluids amount | 1.000(1.000-1.000;*P*=0.004) | 1.000(1.000-1.000;*P*=0.002) |
| Out fluids amount | 1.000(0.999-1.000;*P*<0.001) | 1.000(0.999-1.000;*P*<0.001) |
| Erythrocyte transfusions | 2.016(1.540-2.632;*P*<0.001) | 1.931(1.542-2.413;*P*<0.001) |
| Hemorrhage | 1.000(1.000-1.001;*P*<0.001) | 1.000(1.000-1.001;*P*<0.001) |
| Vasoactive drugs | 1.856(1.419-2.421;*P*<0.001) | 1.925(1.534-2.407;*P*<0.001) |

*AKI* acute kidney injury, *IOH* intraoperative hypotension, *MAP* mean arterial pressure, *ASA* American Society of Anesthesiologists,*RASI*renin-angiotensin system inhibitor

Table S4. VIF of different factors

| **Variables** | **VIF** |
| --- | --- |
| Age | 1.279 |
| Sex | 1.153 |
| Basic MAP | 1.208 |
| Smoke | 1.640 |
| Alcohol | 1.583 |
| Diabetes | 1.025 |
| Emergency | 1.328 |
| ASA grade | 1.501 |
| **Preoperative** |  |
| Diuretics | 1.210 |
| RASI | 1.125 |
| Calcium antagonists | 1.050 |
| β-Blockers | 1.092 |
| Albumin | 1.266 |
| Hemoglobin | 1.383 |
| Alanine aminotransferase | 1.035 |
| Magnitude of surgery | 1.098 |
| Type of anaesthesia | 1.471 |
| Duration of surgery | 2.309 |
| In fluids amount | 2.942 |
| Out fluids amount | 1.624 |
| Erythrocyte transfusions | 1.672 |
| Vasoactive drugs | 1.209 |

*VIF* variance inflation factor, *MAP* mean arterial pressure, *ASA* American Society of Anesthesiologists, *RASI* renin-angiotensin system inhibitor
